# An Exploratory Analysis of Fifteen Years Suicide Trends Using Population-Level Data From Croatian Committed Suicides Registry

**DOI:** 10.3389/fpubh.2022.857284

**Published:** 2022-05-30

**Authors:** Vanja Pajić, Stjepan Orešković

**Affiliations:** ^1^Independent Researcher, Zagreb, Croatia; ^2^Department of Medical Sociology and Health Economics, School of Medicine, University of Zagreb, Zagreb, Croatia

**Keywords:** committed suicide, suicide rate, Death Certificate, incidence, regional distribution

## Abstract

**Objectives:**

The provide a descriptive analysis of the available population-level aggregated data on committed suicides in the Republic of Croatia, in the 2004–2018 period, showing emerging trends in suicide incidence focusing on sex/age/geographical distribution of suicides and the primary and secondary causes of suicide mortality, as well as making comparisons with similar neighboring neighboring countries.

**Methods:**

The aggregated suicide data were obtained from the Croatian Committed Suicides Registry, a national registry maintained by the public health authority. The raw data extract was organized into tables according to several variables (age, sex, place of birth, place of residence, and cause of death). Simple descriptive statistics were performed on the structured data.

**Results:**

Despite being among the highest in the world and EU, the number of committed suicides in Croatia is in decline since 2004. A higher number of suicides by males was observed when compared to females. Most of the suicides occur during spring and summer. The wealthier, northern continental region of the country had the highest average rate of committed suicides per 1,00,000 population, contrary to some of the findings in the published literature associating economic instability with suicide. The most common way to commit suicide for both sexes in all age groups is self-harm by hanging, strangulation and suffocation. Suicide by firearm and explosive devices discharge remains higher than the global average.

**Conclusions:**

Despite its steadily declining incidence rates in the past 20 years, suicides remain a major public health challenge in Croatia. Results may bolster the Ugro-Finnish suicide hypothesis, linking higher suicide rates to regions with populations of Hungarian descent.

## Introduction

Suicides are one of the leading causes of death from injuries in the Republic of Croatia. As a major public health issue, suicide remains a growing national concern with the suicide rates in Croatia being among the highest in the world. In 2016, Croatia was the 16^th^ highest ranking country globally and 8^th^ in the EU when it came to suicide with an average overall (both male and female) suicide rate of 16 per 1,00,000 population (1, 2). Moreover, when compared to other causes of death in Croatia, death by suicide is ranked among the top 10 most frequent causes of death (2).

Suicide remains an extremely complex phenomenon that includes the interactions of factors at different levels of analysis: the systemic level (access to suicide means such as post-war firearms and ammunition), societal level (socio-economic determinants of mental health), community level (support from the community, family, and peer group) and individual level (sex, age, medical and biological determinants).

Despite this, little comprehensive epidemiological research has been made regarding the population-wide study of suicide in Croatia in the 21^st^ century that would account for these different factors. Some noteworthy examples do exist, mostly focusing on specific demographics. Notably, a number of studies have addressed the 1990's post-war suicide as a direct result of the trauma experienced by many people during the Croatian War of Independence in the early 1990s (3–10).

Also, several studies have explored the epidemiological characteristics of suicide in specific Croatian regions (11–15) or were otherwise focusing on a specific demographic group characterized by age (16–20) and the relevant diagnosis (21) and therapy (22). Additionally, several papers analyzing epidemiological data on suicide in Croatia do exist (23–25), however, there seems to be lack of research focusing on the entirety of the Croatian population in the last 20 years.

This seems surprising considering that Croatia has one of the highest suicide rates globally and within the EU, only being comparable to other countries in the same geographical region, such as Slovenia and Hungary. The reason of such high suicide rates in the region are not fully understood, however several attempts were made to indicate a link between genetics and suicide ideation in Ugro-Finnish populations from Finland and the Baltic countries, including Russia, Belarus, and Ukraine all the way to eastern Central Europe and the Western Balkans – including Croatia (26–28).

The aim of the research is to describe some of the key factors contributing to the incidence of suicides in all the territory of the Republic of Croatia, based on the aggregated data obtained from the Croatian Institute of Public Health, and focusing on the period from 2004 to 2018. Root causes of suicide and suicide ideation are out of scope in this research, however Croatia is compared to other neighboring countries with similar geographical position, economic status, as well as age and sex distribution, adding to the body of research on suicides in the broader region, hopefully bolstering the main assumptions regarding the Ugro-Finnish suicide hypothesis.

Our population of interest were the persons who committed suicide on the territory of Croatia, between 2004 and 2018 (including the years 2004 and 2018), which were registered in the Croatian Committed Suicides Registry.

## Methods

The source of data in the Croatian Committed Suicide Registry is the Death Certificate. The Death Certificate is an official medical document issued by a doctor or any other person authorized by law to determine death, or a health care institution if the person died in such an institution. The method of data collection is determined by the Annual Implementation Plan of Statistical Activities of the Republic of Croatia under the heading of Statistics on Psychosis with the Register for Psychosis and the Register of Suicides (29). This registry collects data on suicide completions and therefore suicide attempts were left out of scope.

The suicide data from the Croatian Committed Suicide Registry was retrieved as a Microsoft Excel sheet export from the Croatian Institute of Public Health's database which included the raw cumulative number of suicides cases on the territory of the Republic of Croatia, during the 15-year period between 1^st^ January 2004 and 31^st^ December 2018, aggregated monthly (January-December) for each year separately, according to:

*a) The county of birth (Croatia)*,
*b) The county of residence (Croatia)*,
*c) Age (expressed in age groups 0*–*19, 20*–*39, 40*–*59, 60*–*79, 80*–99), (*ages above 99 were omitted due to an extremely low number of reported cases)
*d) Sex (Male and Female, respectively), and*
*e) The proximal and external cause of death (expressed as WHO ICD-10 codes: S00*–*T98 for Injury, poisoning and certain other consequences of external causes; and X60*–*X84 for Intentional self-harm), (the ICD-10 codes for Event of undetermined intent (Y10*–*Y34) and especially Y34 for External cause of death of undetermined intent were excluded from this analysis due to a low number of reported cases related to a rigorous process of data clean-up during the suicide registration process)*.

To test our main assumption about the normal distribution of the available aggregated data, the data was first organized into tables according to the parameters outlined above. Data was cleaned up by removing incorrect, corrupted, incorrectly formatted, duplicate, or incomplete entries. Simple descriptive statistics were performed on the data. Our final dataset included a total of 11,405 cases of suicide in the Republic of Croatia, during the time period between January 1^st^ 2004 and December 31^st^ 2018. The age-standardized mortality rate was taken as a weighted average of the age-specific suicide rates per 1,00,000 persons in the Croatian population each year.

In an attempt to make the analyzed data comparable with other countries in the region, a simple descriptive statistic of the data using the main output variable (i.e., the number of committed suicides from the national committed suicides registry) was then calculated for each year and the complete dataset. The mean for the complete dataset was set at 63.36 with the Standard Error of 0.91, r approximating a normal distribution of our values and implying a smaller spread of data points across the dataset. The median was calculated at 62 and the mode at 65, which confirms this. Standard deviation was set at 12.23, indicating that our extreme data points are close to the mean considering the entire dataset. The slope of the suicide rate trends from 2004 to 2018 was calculated at −0.3 with an R value of 0.81.

Under the null hypothesis of normality, the test statistic Jarque-Bera (JB) follows a Chi-Square distribution with 2 degrees of freedom. Thus, to find the *p*-value for the test we used the following function in Excel: = CHISQ.DIST.RT. Our *p*-value was calculated at *p* = 0.21314 (i.e., > 0.05), and therefore, we can accept the hypothesis of normality when applied to our dataset. In other words, the distribution of difference scores may have varied slightly from month to months, and – less severely - year to year but could be fitted to a normal distribution. A stationary time series is one whose properties do not depend on the time at which the series is observed. Thus, time series with trends, or with seasonality, are not stationary — as the trend and seasonality will affect the value of the time series at different times. We could conclude that our time were are not stationary, i.e., we have observed a trend in the time series which corresponds to our initial expectations. The Augmented Dickey-Fuller tests were performed for all selected variables and demonstrated no presence of a unit root in the respective datasets.

Data was analyzed for any significant patterns considering age and sex distribution, seasonality and geographical factors. Additionally, primary and secondary causes of suicide were analyzed in order to research any suicide completion trends in the past 20 years.

## Results

**Figure 1:**
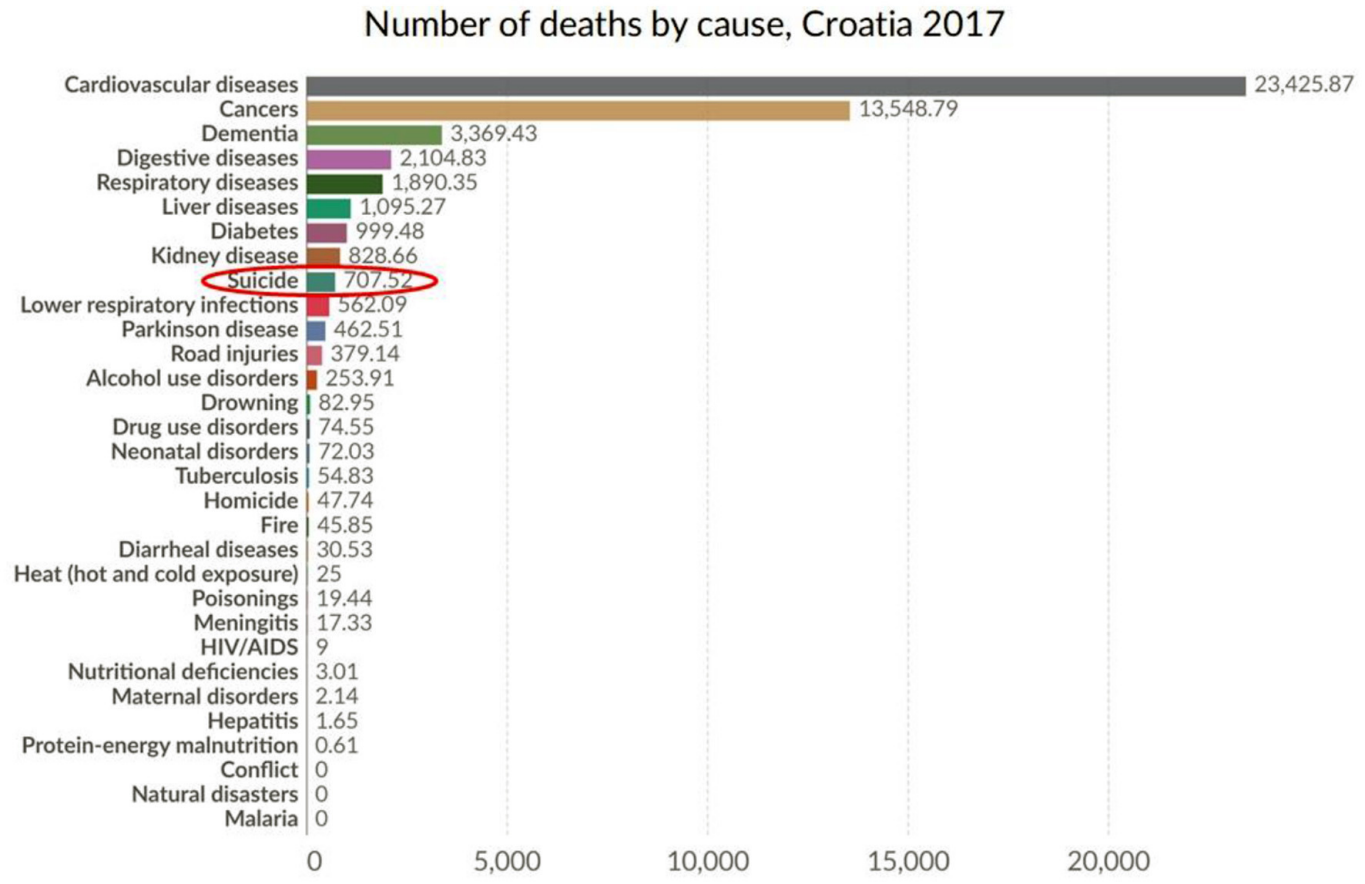
Number of deaths by cause, in Croatia, 2017 (Croatia, 2021). Source: IHME, Global Burden of Disease (GBD). OurWorldInData.org/suicide.

Suicide is the 8^th^ leading cause of death in Croatia, after cardiovascular diseases, cancers, dementia, digestive diseases, respiratory diseases, liver diseases, diabetes, and kidney diseases (see Figure 1), and resulting in twice as much deaths as road accidents.

Despite of this, suicide still presents a serious public health issue that has been poorly dealt with during the past 20 years in Croatia. Firstly, there has been a lack of studies aimed at understanding social determinants of health. Secondly, research focusing on specific demographics at risk of suicide was traditionally focused on war victims and veterans with PTSD, thus neglecting other vulnerable groups such as teenagers and the elderly. The results presented here will identify age and sex-specific aspects of suicide in Croatia, as well as seasonal patterns of suicide, the differences in suicide rates between the continental and coastal regions of the country, and the preferred suicide methods.

Between 2004 and 2018, there was a total of 11,405 suicide cases in Croatia (11,405 data points from our data analysis) in a population of over 4 million (4,304,600 in 2004, and 4,087,843 population in 2018), or 760 cases per year on average, which gives a suicide rate of 18 per 1,00,000 population. The overall decreasing trend is clearly identifiable through this period, with a 18% decrease in suicide rates when comparing 2004 to 2018. Age-standardized suicide rates per 1,00,000 population decreased from 20 in the early 2004 to 17 in the 2018.

Despite the declining trend of suicide cases since the early 90s throughout the region, suicide rates have been traditionally high in surrounding countries (Hungary, Slovenia, and Serbia), and remain higher than in other EU countries.

**Figure 2:**
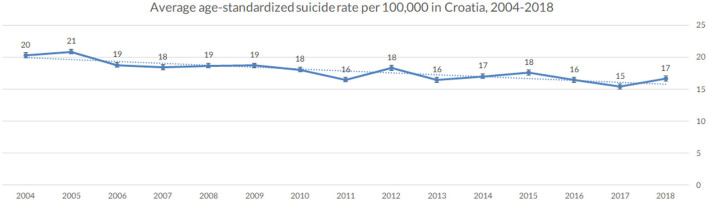
Age-standardized suicide rate per 100,000 population, in Croatia (averaged across the observed period), 2004–2018 (Croatia, 2021).

**Figure 3:**
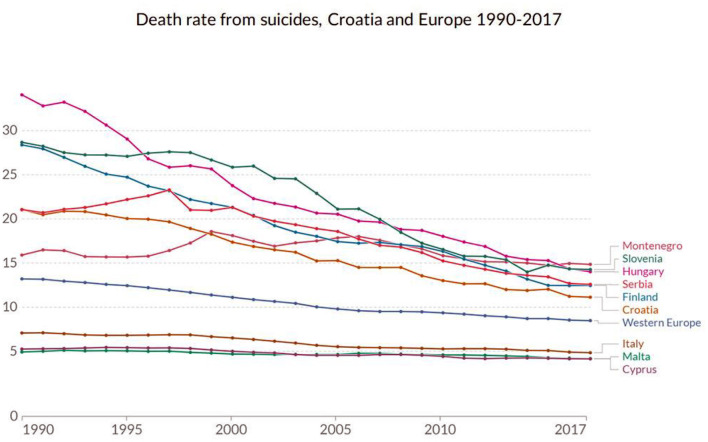
Death rate from suicides, Croatia, with neighboring (Hungary, Slovenia, Serbia and Montenegro), selected Mediterranean countries (Italy, Malta, Cyprus), Finland (pointing toward the Ugro-Finnic suicide hypothesis), and the Western Europe, 1990–2017 (Croatia, 2021). Source: IHME, Global Burden of Disease (GBD). OurWorldInData.org/suicide.

Suicide rates in Croatia (per 1,00,000) were highest in the following years (see Figure 2): 2005 (21), 2004 (20), 2006, 2008 and 2009 (19). These were followed by 2007, 2010 and 2012 (18), 2014 (17) and 2011 (16). The years with the lowest suicide rates were 2018 (17), 2013 and 2016 (16), and 2017 (15), respectively. Within Europe, Baltic countries are at the forefront of suicide incidence, with Hungary, Slovenia and Croatia being close behind, with suicide rates up to 15+ per 1,00,000 inhabitants, well above the EU27 average (see Figure 3).

### Suicide and Sex in Croatia, 2004–2018

A higher average number of suicide cases was observed in the male population when compared to females, which is consistent with both the global and earlier national trends. On average ¾ of all suicides in Croatia were committed by men. It is worth noticing that this male to female ratio of suicide cases has been decreasing in the past 15 years, with the 3.5:1 relationship in 2004 and 2.82:1 in 2018.

### Suicide and Age in Croatia, 2004–2018

**Figure 4:**
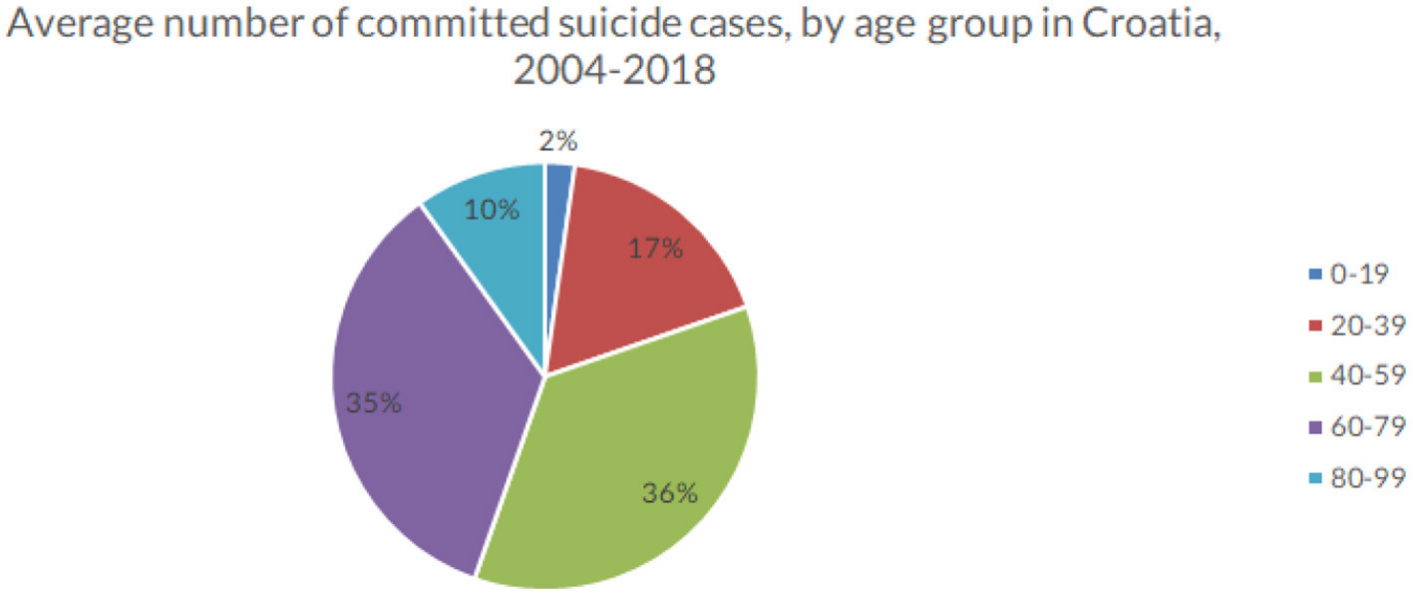
Average number of suicide cases by age group, in Croatia (averaged across the observed period), 2004–2018 (Croatia, 2021).

The majority (88%) of committed suicides in Croatia occurred between the ages of 20–79 (see Figure 4). Within this broader age group, 17% of all suicides were committed between the ages of 20 and 39, 36% between the ages of 40 and 59 and 35% between the ages of 60 and 79. A slight decline in suicide rates in all ages except in the 0–19 and 80–99 age group was observed. Although the average number of committed suicide cases for both sexes has decreased overall, the age-adjusted suicide rates for men and women increased with age.

### Suicide and Seasonality

**Figure 5:**
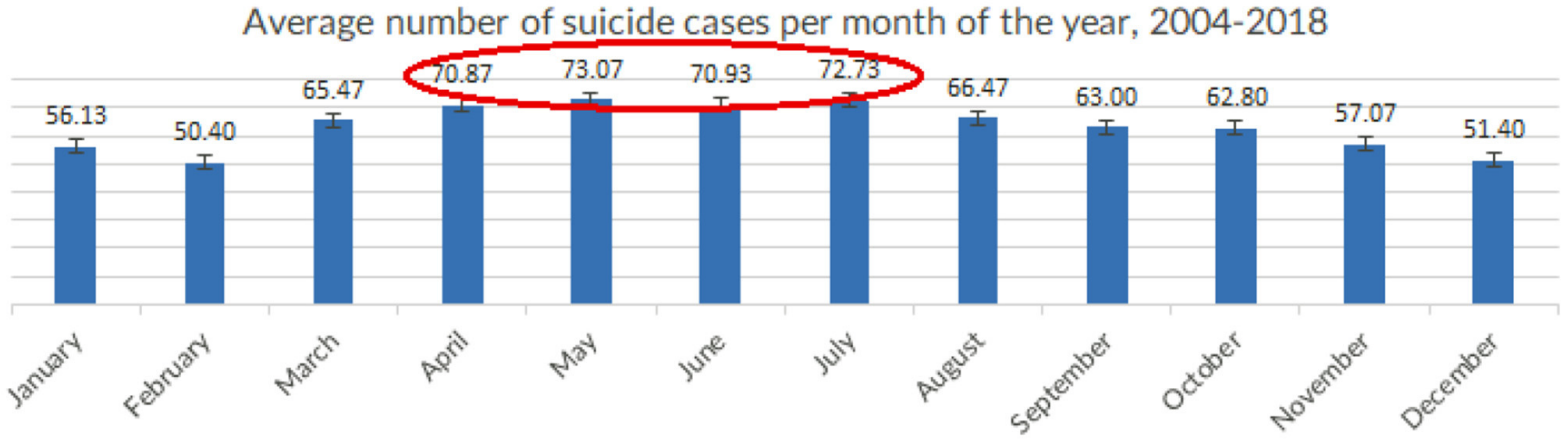
Average number of suicide cases per month of the year, in Croatia (averaged across the observed period), 2004-2018 (Croatia, 2021).

In Croatia, most suicides were committed during spring and summer, with a surge in suicide rates beginning in March and continuing through May, June and July and then starting to decline in August and September, continuously dropping in autumn and winter (see Figure 5). The highest average number of committed suicides from 2004 to 2018 was observed in May (73), followed by July (72), June (71) and April (70) respectively. The lowest average numbers of committed suicides occurred in February (50) and December (51), closely followed by January (56) and November (57), with other months falling somewhere in between – March (65), August (66), September (63) and October (62), suggesting a seasonal pattern of committed suicides. This seasonality of suicides was observable through the entire studied period and remains constant throughout the years.

### Suicide and Place of Birth

**Figure 6:**
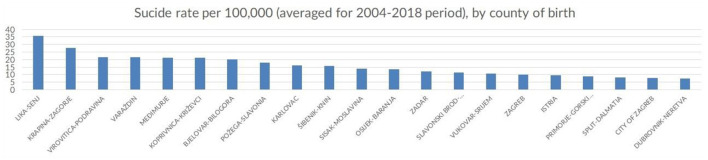
Number of suicide cases per county of birth, in Croatia, (averaged across the observed period), 2004-2018 (Croatia, 2021).

**Figure 7:**
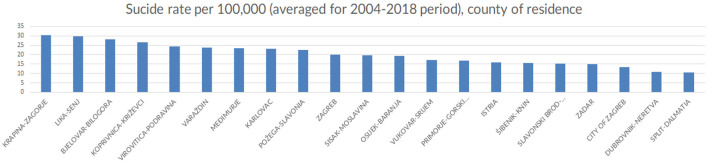
Number of suicide cases per county of residence, in Croatia, (averaged across the observed period), 2004-2018 (Croatia, 2021).

When looking at the 21 of Croatia's counties differences were observed in suicide rates across the country, accounting for the county of birth as well as the county of residence (see Figures 6, 7). The highest average suicide rate for any county was observed in Lika-Senj county (36) and the lowest in Dubrovnik-Neretva county (8). Overall, the lower suicide rates were observed in coastal counties: Istria (10), Primorje-Gorski kotar (9), and Split-Dalmatia (8). Šibenik-Knin county and Zadar were an exemption to this with an average of 16 and 12 suicide cases per 1,00,000 population. The northern continental part of the Country had the highest suicide rates with Krapina-Zagorje (28), VaraŽdin (22) and Medimurje (21) being among the top five. The City of Zagreb which is populated with about ¼ of the nation's population had a lower suicide rate then most counties on average (10). This clustering of suicide cases in persons coming from specific locations was not extensively researched in Croatia. Moreover, it is worthwhile comparing this data with that of suicide cases related to the place of residence to identify possible causative patterns.

### Suicide and Place of Residence

With regards to the place of residence, the highest age-standardized suicide rates were observed in the continental parts of the country, in Krapina-Zagorje (30), Lika-Senj (30), closely followed by Bjelovar-Bilogora (28) and Koprivnica-KriŽevci (27). Similarly, lower suicide rates were observed in the counties located in the coastal region, in Split-Dalmatia (10), Dubrovnik-Neretva (11) and Zadar (15) counties. Again, the City of Zagreb was at the bottom of the list with one of the lowest average suicide rates in the country (13).

### Suicide Methods / Causes of Death

**Figure 8:**
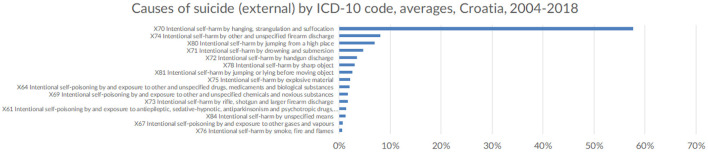
Causes of suicide (external) by ICD-10 code, in Croatia (averaged across the observed period), 2004-2018 (Croatia, 2021).

From 2004 to 2018, the most common way to commit suicide in both sexes for all groups was intentional self-harm by hanging, strangulation and suffocation (X70) i.e., suicide by hanging (57.71%) (see Figure 8). The impact of hanging on suicide rates is replacing that of intentional self-harm by other and unspecified firearm discharge (X74), i.e., suicide committed by shooting a firearm (8.09%), and is closely followed by intentional self-harm by jumping from a high place (X80), i.e., suicide committed by jumping of a building or bridge (6.96%). Regarding proximal causes of suicide, asphyxiation (57.43%) is the primary cause of death by suicide which is associated with hanging as the major suicide completion method. Open wound of head (9.58%) is the second most frequent proximal cause of death which is consistent with the finding that the intentional self-harm by other and unspecified firearm discharge, i.e., shooting oneself in the head is the second most prominent suicide method in Croatia in the observed period. Unspecified multiple injuries come at third place (7.89%) which might be consistent with the finding about intentional self-harm by jumping from a high place as the third most preferred suicide method.

## Discussion

Suicides are one of the leading causes of death from injuries in Croatia. After the years following the Croatian War of Independence (1991 to 1995), there has been a downward trend in the number of suicides, however suicide rates remain to be one of the highest in Europe and the world. Both male and female suicide rates dropped significantly during the 15-year period. Suicide rates for persons under 19 and above 80 have not seen the same decline in suicide rates as did other age groups. The biggest challenge for the healthcare system in Croatia will be to sustain this general trend of suicide rate decline in the longer run, considering that the overall population is aging, and suicide rates appear to increase (or at least no decline) with age.

Considering comparable suicide rates in neighboring countries in which war did not occur in recent history (e.g., Hungary), the main assumption is that the longer-term impact of war on suicide can be considered as marginal. Counties mostly affected by war in the early 1990s were amongst the ones with the lowest average number of sucides in the studied period.

Although a variety of different variables influences suicide, from biological, psychological to socio-economic, it is well-established that suicide rates are the highest in former socialist and communist countries, from Baltics and the Russian federation in the north, to south-east Europe and the Western Balkan countries (30, 31).

According to available data on the EU countries for comparison, suicide rates were lowest in Cyprus, Greece, Italy, and Malta (all being Mediterranean countries), where there were < six suicides per 100,000 population in 2017. In contrast, Lithuania and Slovenia had the highest suicide rate in the same year, with 26 and 20 deaths per 1,00,000 population, respectively. Croatia was following closely with 15 committed suicide cases per 1,00,000, the lowest suicide rate in the studied 2004–2018 period, possibly bolstering the Ugro-Finnish suicide hypothesis of suicide clustering in countries with population of Hungarian descent.

Emerging trends regarding suicide in Croatia demonstrate that the male to female ratio of committed suicide has been decreasing in the past 15 years, making women almost as likely to commit suicide as males in the late 2010s. Additionally, suicide rates have been declining in the age group comprised of war veterans (approximately 40–60 years old) while increasing in younger and older generations (0–19 and 80–99 years old). The sex distribution pattern of suicide is consistent with the findings from other countries in the region, as well as western European and Mediterranean countries (32, 33). Unfortunately, the data that we analyzed did not give us insight into the gender differences in different age groups. However, some research suggests that the male to female suicide ratio increased in older age groups (34).

Within this broader age group, 17% of all suicides were committed between the ages of 20 and 39, 36% between the ages of 40 and 59 and 35% between the ages of 60 and 79. A slight decline in suicide rates in all ages except in the 0–19 and 80–99 age group was observed. Although the average number of committed suicide cases for both sexes has decreased overall, the age-adjusted suicide rates for men and women increased with age. These results are consistent with findings across Europe and the world and will continue to present a challenge as populations continue to grow older (35, 36).

Seasonal patterns are a known factor for suicide risk, and the seasonal variation of deaths by suicide offers an important pathway in the study of possible suicide determinants of suicide and consequently suicide prevention (37). This seasonal distribution of suicide is following a phenomenon that was observed by previous studies around the world yet remains poorly understood (38).

Interestingly, the findings from the analysis of county-wide distribution of suicide rates are inconsistent with similar research pointing at various economic reasons impacting suicide rates such as unemployment (39), as the regions in Croatia in which the highest suicide rates were observed traditionally have the lowest unemployment rates. Moreover, there were no noticeable peaks in suicide rates that could be associated with the global financial crisis of 2008, as was suggested by some previous research (40).

Average age-standardized suicide rates were relatively lower in the more densely populated regions, most notably in the counties containing Croatia's four largest cities – Zagreb, Split, Rijeka and Osijek), despite being the parts of the country with the biggest income inequality. This is also pointing to suicides being committed more often in urban as opposed to rural environments. Furthermore, the counties situated along the Mediterranean coastline had noticeably lower suicide rates that richer and more industrially developed northern regions on the continent. This was the most surprising finding of this research which does not follow similar trends in other European countries. This geographical determinant of suicide seems even more significant considering that the observed suicide rates were similar regardless of whether one was considering the specific location as the county of birth or county of residence. Notably, this research did not study the migratory patterns between counties and the impact of moving from one county to another on suicide cannot be estimated. The data from the committed suicide registry was aggregated on the county level in an attempt to safeguard personal information from being indirectly and invertedly revealable in case of e.g., villages and smaller towns with only a single completed suicide in any given time period.

Seasonal patterns of suicides have been demonstrated in many countries, with most research identifying spring and summer peaks. Despite Croatia's favorable geographic position in the east Adriatic, with mild continental climate inland and Mediterranean climate along the coast, the seasonality of suicides follows that of e.g., Baltic countries, with most suicides occurring through the warmest months of the year – from late spring to high summer regardless of the region (i.e., continental, or coastal).

Finally, the huge proportion of suicides is still caused by firearm discharge, arguably due to the presence of weapons in many households as a legacy of the recent war during the early 1990s. Curiously, there is still a fairly large number of suicides committed by the intentional activation of explosive devices (such as mines and hand grenades), which is one of the more peculiar suicide methods that is specific to Croatia, likely to be associated with the war as well. There is an increasing trend of hanging by the neck as the preferred method of suicide, followed by jumping from high places or in front of a moving object. Self-poisoning by overmedication of ingestion of a harmful substance, as well as self-harm by a sharp object remain lower on the list of suicide methods and account for little more than 3% of all committed suicides, unlike most other western European countries. Suicide from self-poisoning by means of pesticide ingestion or inhalation is low which is consistent with similar findings from EU countries (41) which is most likely due to bans on the use of dangerous pesticides and wide-spread pesticide-control in general. Unlike other EU countries, Croatia has a higher number of suicides committed by the use of guns, with similar findings from the USA highlight the correlation between firearms possession and firearm suicides (42). As the government enforces new gun control measures in future years, we might expect a lower number of committed suicides using firearms and other banned explosive devices. Death by jumping from a high place seems to be on the rise, replacing the use of firearms as the preferred suicide method, despite the relative lack of tall buildings throughout the country.

Due to the lack of interoperability between various healthcare databases, e.g., those containing hospital records of suicide victimsand the national committed suicides registry, it is difficult to draw any conclusions between mental health issues and suicide completion. Future studies may draw conclusions from similar research in neighboring countries with similar suicide trends.

Despite the vast amount of suicide data that is regularly and systematically collected by the Croatian Institute of Public Health, there remains a large potential for data exploration and analysis of the Croatian Committed Suicide Registry. Research on geographical location regarding suicide should provide additional information on suicide clustering in continental regions of the country. The peculiar finding of richer regions having more suicides needs to be explored as well, as this doesn't fit well with the international research on the socioeconomic determinants of suicide. Data on in-county migrations between regions (continental and coastal) and individual counties may provide insight into specific geographical determinants of suicide. It is worth mentioning, however, that the counties with the highest suicide rates in Croatia border Slovenia and Hungary, the two countries with some of the highest suicide rates in the world which would be a finding worth further exploring.

The outcome of this and future studies in suicidality may be used to inform and support public health suicide prevention policies which have been seriously lacking in Croatia. We propose a location-sensitive approach that takes into account the clustering of suicides in the continental, northernmost parts of the country. This is especially interesting in the light of the Ugro-Finnish suicide hypothesis, linking higher rates of suicide to people of Hungarian ancestry, that are typically clustered in the norther parts of Croatia.

Future research may also consider the seasonal pattern of suicides during late spring and summer months, declining importance of using firearms and explosives as the preferred suicide method, and the shifting trends of suicide cases according to age and sex, as these may unearth country-specific aspects of suicide not found in other countries in the region.

### Limitations of This Study

The data for this study was analyzed at both the yearly and monthly aggregation levels. The study covered only those suicide cases that have been officially documented in the national suicide registry and does not account for non-reported suicides or suicides potentially misclassified as undetermined deaths. Moreover, this study focuses only on committed suicides and cannot make assumptions regarding attempted suicides. The data analysis showed only a negligible number of cases reported as deaths of unspecified intent or origin (<0.05%) which were omitted from this study. The national suicide registry only records committed suicides and attempted suicides were left out of scope in this research, making any conclusions regarding the prevalence of suicide attempts leading to suicide completion impossible. In future studies, it would be interesting to see how population demographics influence suicide attempts and what impact suicide ideation has on suicide completion.

During the course of drafting this paper, it came to the attention of authors that a more detailed analysis should be made in comparing countries in the Ugro-Finnic suicide belt reaching from Finland, the Baltics, Eastern Europe and the Balkans, with Croatia. This will be explored in a future paper.

## Conclusions

Croatian suicide trends in regard to sex and age are similar to that of other EU countries, making the effect of 1990s war on suicide likely negligeable at the population level.

Effects of seasonality were found in suicide rates with a peak in summer months. Seasonal effect remained unchanged after adjusting for age and sex.

Suicide rates are higher in the northern regions of the country, closer to Slovenia and Hungary, pointing to a possible connection with the Ugro-Finnish suicide hypothesis.

Suicide methods differ somewhat from other EU countries, with high number of suicides committed by firearms, likely due to a large number of weapons in households after the 1990s war.

## Data Availability Statement

The data analyzed in this study is subject to the following licenses/restrictions: Access to dataset was approved as part of the PhD research by the article's principle author. The data was extracted in an aggregated format. Requests to access these datasets should be directed to https://www.hzjz.hr.

## Author Contributions

VP: PhD researcher, principal investigator, data analyst and article principal author. SO: PhD research mentor. All authors contributed to the article and approved the submitted version.

## Funding

This research was self-funded by the authors.

## Conflict of Interest

The authors declare that the research was conducted in the absence of any commercial or financial relationships that could be construed as a potential conflict of interest.

## Publisher's Note

All claims expressed in this article are solely those of the authors and do not necessarily represent those of their affiliated organizations, or those of the publisher, the editors and the reviewers. Any product that may be evaluated in this article, or claim that may be made by its manufacturer, is not guaranteed or endorsed by the publisher.
